# Triamcinolone-loaded nanocarriers: a novel strategy to mitigate cognitive and emotional sequelae induced by traumatic brain injury via modulation of oxidative stress

**DOI:** 10.3389/fnbeh.2025.1638417

**Published:** 2025-08-12

**Authors:** Aída Marcotti, Sofía De la Fuente, María Lina Formica, Agustín Jorge Montivero, Martina Ramires, Verónica Leonor Romero, María Florencia Constantin, María Jazmín Silvero, María Cecilia Becerra, Gastón Diego Calfa, Miriam Beatriz Virgolini, Santiago Daniel Palma, Mariela Fernanda Pérez

**Affiliations:** ^1^Universidad Nacional De Córdoba, Facultad De Ciencias Químicas, Departamento De Farmacología Otto Orshinger, Córdoba, Argentina; ^2^CONICET, Instituto De Farmacología Experimental De Córdoba (IFEC), Córdoba, Argentina; ^3^Universidad Nacional De Córdoba, Facultad De Ciencias Químicas, Departamento De Ciencias Farmacéuticas, Córdoba, Argentina; ^4^CONICET, Unidad De Investigación y Desarrollo En Tecnología Farmacéutica (UNITEFA), Córdoba, Argentina

**Keywords:** traumatic brain injury, oxidative stress, neuroinflammation, cognitive deficits, triamcinolone-loaded lipid nanocapsules

## Abstract

**Introduction:**

Traumatic brain injury is the leading cause of death and disability in individuals under 40 years old. It induces various neuropathological outcomes, including cognitive, emotional, and physiological deficits, likely linked to early neuroinflammatory processes. In an animal model, mild traumatic brain injury (mTBI) has been shown to elevate oxidative stress biomarkers, such as advanced oxidation protein products (AOPP) and malondialdehyde (MDA), which persist for over a week. Despite extensive research on anti-inflammatory and neuroprotective therapies, most preclinical and clinical studies report limited efficacy. Synthetic glucocorticoids offer potential for early treatment of TBI-induced neuroinflammation, but clinical use is hindered by adverse effects and poor central nervous system biodistribution. Triamcinolone possesses anti-inflammatory, anti-angiogenic, and microglial inhibitory properties, although it has poor solubility and limited blood-brain barrier (BBB) penetration. Lipid nanocapsules (LNCs) may enhance TR solubility, bioavailability, BBB permeation, and intracellular delivery. This study aimed to evaluate the efficacy of triamcinolone-loaded LNCs (NT) on oxidative stress and cognitive-emotional outcomes following mTBI.

**Methods:**

Adult male Wistar rats were subjected to closed-head mTBI via a 45 g weight-drop method, under anesthesia. Animals received NT, conventional triamcinolone, or empty LNCs, 15 minutes and 24 hours post-injury. They were sacrificed 24 hours, 1 or 7 days later for biochemical analysis of AOPP, MDA, and antioxidant enzymes (catalase and superoxide dismutase) activity in the hippocampus, prefrontal, and motor cortices. Separate cohorts underwent behavioral tests assessing memory (novel object recognition, Y-maze, and fear conditioning), 7 days after mTBI.

**Results:**

mTBI induced significant impairments in recognition memory and fear retention, as well as increased AOPP, MDA, and CAT activity. SOD levels peaked at 24 h and normalized by day 7. NT, but not conventional TR, effectively prevented behavioral deficits and normalized OS markers. Importantly, early NT treatment reduced CAT overactivation at 7 days.

**Discussion:**

This study provides the first evidence of the efficacy of NT in mitigating cognitive and emotional sequelae following mTBI, likely through enhanced brain delivery and early modulation of oxidative stress pathways.

## 1 Introduction

Traumatic brain injury is a leading cause of death and disability worldwide, particularly among the young population (Dewan et al., 2018; Maas et al., 2022). Mild TBI (mTBI), defined as a Glasgow Outcome Scale score of 13–15 (the most widely used instrument to assess global disability and recovery after TBI), accounts for over 90% of TBI cases presenting to hospitals. This common neurological condition can lead to early cognitive and emotional impairments. The most affected cognitive domains include attention, executive function, working memory, recognition memory, and language (Wylie et al., 2015). Emotional disturbances, such as anxiety and mood disorders, are also frequently reported (Kennedy et al., 2007; Stocchetti and Zanier, 2016; Sanders et al., 2025). These deficits are often accompanied by disruptions in emotion regulation and behavior, collectively referred to as post-concussive dysexecutive syndrome (PCS). Notably, half of adult patients with mTBI do not recover to their pre-injury levels of health within 6 months, and there is a lack of evidence to inform the treatment of these patients (Maas et al., 2022). PCS can occur in patients affected by TBI, regardless of its severity (Arciniegas et al., 2002; Montivero et al., 2021a). More than 50% of mTBI patients experience cognitive deficits 3 months after their injury, and many continue to show functional limitations even 12 months post-injury (Li et al., 2020). Common impairments include reduced work capacity, social function problems, and family disruptions, which significantly impact patients’ quality of life and social functioning.

The pathophysiology of TBI involves primary and secondary injury. Primary injury encompasses damage directly resulting from the mechanical forces of trauma and is predominantly focal, such as skull fractures, epidural, subdural, or intraparenchymal hematomas, and cerebral lacerations. However, it may also manifest diffusely, as seen in diffuse axonal injury at the time of trauma. Secondary injury begins immediately afterward and can persist for days to months. It is characterized by neuroinflammation and increased oxidative stress (OS), both of which contribute to neuronal dysfunction and neurodegeneration, exacerbating cognitive and emotional symptoms. Neuroinflammatory cascades involve the release of pro-inflammatory cytokines, oxidative metabolites, and prostaglandins, leading to lipid and protein peroxidation, cerebral edema, and, ultimately, neuronal cell death (Krishnamurthy and Laskowitz, 2016; Montivero et al., 2021a). Secondary injuries may underlie changes in brain function, revealing disorders of large neural networks involved in cognition and their relationship to PCS among mTBI populations (Li et al., 2020; Chan et al., 2024; Mavroudis et al., 2024). There is growing evidence suggesting that neuroinflammation plays a fundamental role in the long-term sequelae induced by TBI (Pelinka et al., 2004; Farbood et al., 2015). For example, neuroinflammatory serum biomarkers such as glial fibrillary acidic protein (GFAP) and S100B protein have been shown to correlate with injury severity and patient prognosis, as measured by the Glasgow Outcome Scale (Pelinka et al., 2004; Luoto et al., 2017). These biomarkers reflect the extent of astroglial activation, underscoring the central role of neuroinflammation in secondary injury mechanisms after TBI.

Despite understanding the pathophysiological mechanisms behind TBI injuries, effective pharmacological therapies to prevent or reverse secondary injury events remain elusive. Currently, TBI treatment is largely centered on managing cerebral edema, which is challenging to address medically (Donkin and Vink, 2010; Jha et al., 2019). Additionally, some primary care follow-up guidelines for the clinical management of mTBI emphasize proactive screening and treatment for symptoms such as depression, anxiety, insomnia, and headache as core principles (Silverberg et al., 2020).

Synthetic glucocorticoids, such as triamcinolone acetonide (TR), are commonly used for their anti-inflammatory and anti-angiogenic effects, as well as their ability to inhibit microglial activation (Kambhampati et al., 2015). They are particularly utilized in the treatment of ocular inflammatory and neovascular diseases (Ayalasomayajula et al., 2009; Kiernan and Mieler, 2009) and for managing spasticity in multiple sclerosis (Rommer et al., 2016). The use of GCS in treating TBI has a long history (Roberts et al., 2004) due to its capacity to inhibit vascular endothelial growth factor, besides its anti-inflammatory and immunosuppressive activity, a key mediator of blood-brain barrier (BBB) permeability (Murayi and Chittiboina, 2016), which suggests a potential role in reducing cerebral edema. However, clinical studies have yielded mixed results. A large multicenter trial indicated no significant overall benefit, although further analysis revealed improved outcomes and reduced mortality in patients with severe contusions treated with high-dose TR (Grumme et al., 1995). Other studies showed no significant neurological or survival benefits from using dexamethasone or methylprednisolone in TBI (Roberts et al., 2004; Alderson and Roberts, 2005; Hoshide et al., 2016), likely due to limited central nervous system (CNS) penetration. Despite being lipophilic, GCS transport is restricted by BBB efflux proteins like P-glycoprotein and the binding to plasma proteins (Witt and Sandoval, 2014). Experimental and clinical data confirm low concentrations of these drugs in the brain and cerebrospinal fluid following systemic administration (Bannwarth et al., 1997; Meijer et al., 1998). Given their adverse effects in critically ill patients, current guidelines advise against the use of GCS in TBI (Roberts et al., 2004; Alderson and Roberts, 2005; Hoshide et al., 2016), although TR may represent a safer alternative (Umlauf et al., 1983; Grumme et al., 1995).

Despite their therapeutic potential, systemic administration of GCS is limited by side effects. Adverse effects can include neurotoxicity, neuronal apoptosis (Koide et al., 1986; Sapolsky et al., 1990; Buttgereit et al., 1998; Datson et al., 2011; Berger, 2013), and possible cellular damage (Narayanan et al., 2006). Intrathecal administration of TR may lead to arachnoiditis and meningitis (Nelson et al., 1973; Nelson, 1976). Moreover, chronic use of GCS is associated with gastrointestinal risks, including gastritis, ulcers, and bleeding, even when not administered orally (Piper et al., 1991; Oray et al., 2016; Caplan et al., 2017).

Lipid nanocapsules are biomimetic drug delivery systems with a hybrid structure that combines characteristics of polymeric nanocapsules and liposomes (Heurtault et al., 2003). Composed of biodegradable excipients, LNCs exhibit high physical stability in suspension and can achieve prolonged plasma circulation times through controlled particle size (Aparicio-Blanco et al., 2019). They have been employed for targeted drug delivery to the CNS in the treatment of brain tumors (Laine et al., 2012; Hirsjärvi et al., 2014; Groo et al., 2015) and metabolic disorders (Trotier-Faurion et al., 2015). Additionally, TR-loaded LNCs (NT) have demonstrated effectiveness in ocular inflammatory models, reducing inflammation with fewer administrations and side effects compared to standard formulations (Formica et al., 2020). However, the application of NT for systemic treatment of TBI has yet to be explored. Considering all the evidence, the present study aims to assess whether early intervention with NT provides enhanced mTBI outcomes compared to free TR. We propose this approach as a promising strategy for targeting OS induced by mTBI and for reducing cognitive and emotional deficits through improved CNS drug delivery. We anticipate that these findings will contribute to the development of novel therapeutic strategies based on nanotechnology to improve outcomes following brain injury.

## 2 Materials and methods

### 2.1 Ethics

All procedures were conducted following the Guide for the Care and Use of Laboratory Animals as established by the National Institutes of Health (Council, 2011) and had been approved by the Animal Care and Use Committee at the Facultad de Ciencias Químicas, Universidad Nacional de Córdoba (Resolutions Dec. 2336/2019 and 1632/2021).

### 2.2 Animals

Studies were performed in adult male Wistar rats (weighing 280–320 g) obtained from the Department of Pharmacology Otto Orsingher - IFEC - CONICET vivarium (Facultad de Ciencias Químicas, Universidad Nacional de Córdoba, Argentina). Animals were randomly distributed in groups of 4, and housed in plastic boxes with metallic gridded tops, using sterilized sawdust as bedding material without enriching elements. The vivarium was kept at temperature (22 C ± 2°C) and humidity (50% ± 10%)-controlled conditions, and housing rooms were maintained under a 12-h light/dark cycle (lights on at 7 am). Food (mice-rat pellets from GEPSA Grupo Pilar S.A., Argentina) and water (filtered tap water) were available ad libitum. A week before the experiments, animals were handled daily by the operator. All procedures were carried out minimizing the number of animals used and their suffering.

### 2.3 Reagents

Labrafac^®^ WL 1349 was a gift from Gattefosse S.A. (Saint-Priest, France). For free TR suspension, the commercially available suspension from Fortbenton Co. Laboratories was used (Fortcinolona 40, injectable suspension 40 mg/ml). TR for NT preparation was purchased from Pura Química (Córdoba, Argentina). Oleic acid, Kolliphor^®^ HS15, and Lipoid^®^ S75-3 were provided by Sigma-Aldrich (St. Quentin Fallavier, France), BASF (Ludwigshafen, Germany) and Lipoid GmbH (Ludwigshafen, Germany), respectively. Ultrapure water was obtained from the Water Purification System (HF-Super Easy Series, Heal Force, Shanghai, China).

### 2.4 Lipid nanocapsule preparation

The formulations of blank lipid nanocapsules (NV) and NT, with a lipid core composed of a 20% oleic acid, were prepared by the phase inversion temperature process according to a protocol previously described (Heurtault et al., 2002), following the optimized procedure described previously (Formica et al., 2020). For NV, Kolliphor^®^ HS15, Lipoid^®^ S75-3, Labrafac^®^ WL 1349, oleic acid, saline (NaCl 0.9%), and water were mixed under magnetic stirring and submitted to three cycles of heating and cooling between 90°C and 60°C. Then, cold water (2°C) was added to the formulation in the phase inversion zone (75°C). For NT, an exact quantity of TR was first dispersed in a mixture of Labrafac^®^ WL 1349, Lipoid^®^ S75-3, and oleic acid under heating (85°C) and stirring for 1 h. After that, this dispersion was added to a flask containing Kollipor^®^ HS15 and the saline solution, and the three cycles of heating and cooling were carried out as described above, followed by the fast cooling-dilution with ultrapure water. Finally, both formulations were filtered on a 0.22 μm hydrophilic and sterile filter (JetBioFil) under a laminar flow hood.

The size distribution and zeta potential of NV and NT were analyzed using a Zetasizer NanoSerie DTS 1060 (Malvern Instruments S.A., Worcestershire, UK) by Dynamic Light Scattering. For both tests, NV and NT were diluted (1:60) in deionized water, and the average particle size (APS), polydispersity index (PDI), and zeta potential (ZP) were determined at 25°C in triplicate.

### 2.5 mTBI protocol

To model mild human diffuse traumatic brain injury caused by falls or motor vehicle accidents, we utilized Marmarou’s impact acceleration model, which simulates a global impact on the brain (Foda and Marmarou, 1994; Marmarou et al., 1994), with the discrete modifications introduced by Silva Ddos et al. (2011). These modifications aimed to minimize mortality by recreating a mTBI model, applying an impact energy between 0.23 and 0.5 Joules (J), and obtaining animals with normal movement and reflexes after the effects of the anesthetic.

Briefly, animals were anesthetized with ketamine (55 mg/kg) and xylazine (11 mg/kg) and placed on a wooden platform padded with a foam bed. The animal’s head was kept free, but it was positioned beneath one end of a 1-meter aluminum tube, approximately at the midline between Lambda and Bregma. Without surgery, a removable stainless-steel disk was located immediately after the tube, and above the head, to prevent skull fractures. The injury was induced by dropping a 45 g steel sphere down the aluminum tube, delivering an impact with a calculated energy of 0.45 J [0.45 J = 0.045 kg × gravitational acceleration (10 m/s^2^) × 1 m]. Control animals (SHAM group) were anesthetized and placed on the trauma device platform but did not receive the impact (Montivero et al., 2021b).

### 2.6 Experimental groups and drug treatment

#### 2.6.1 Group 1

**FIGURE 1:**
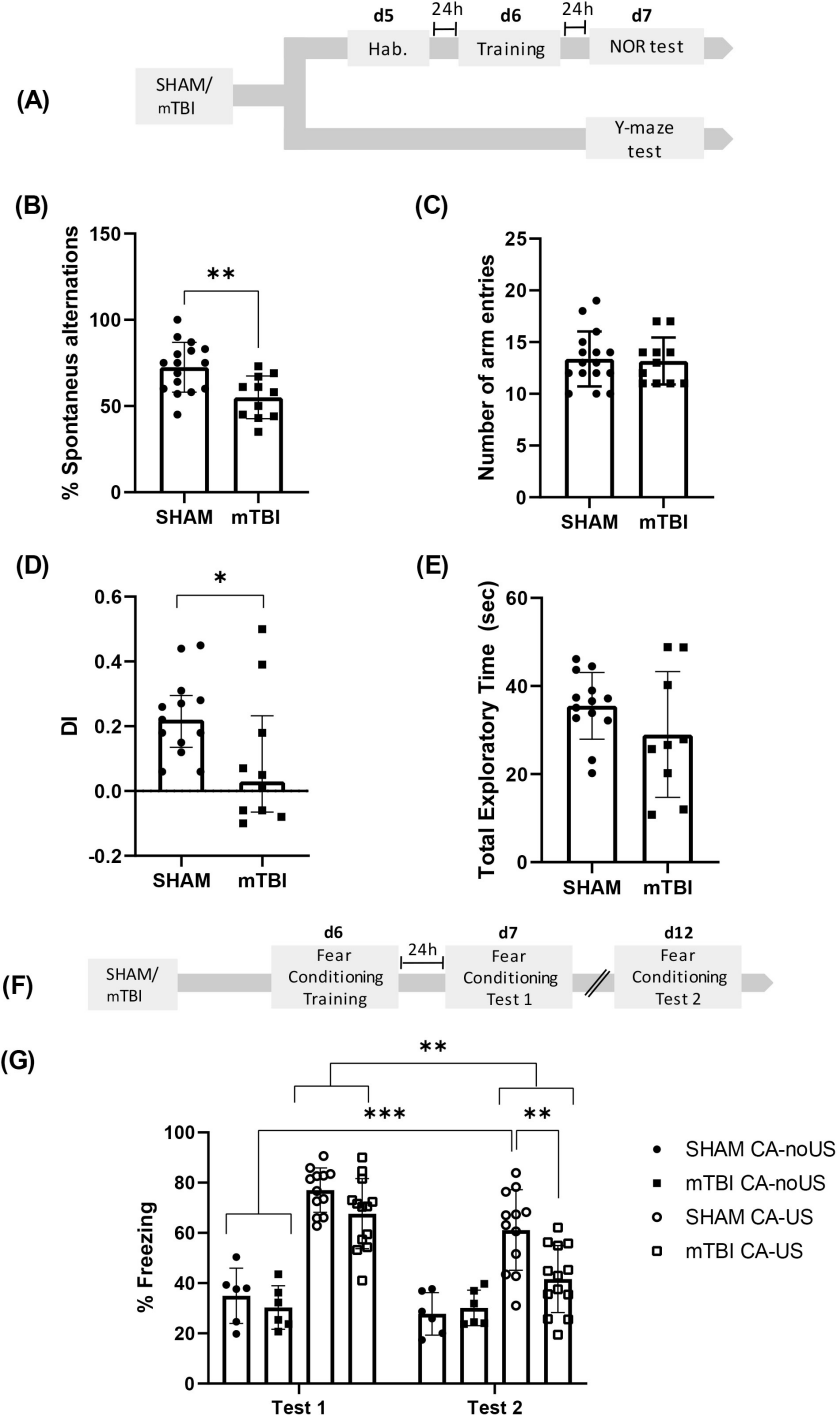
mTBI reduced performance in Y-maze, NOR and fear conditioning retention **(A)** Experimental design for Y-maze and NOR tests after mTBI. Y-Maze test: **(B)** percentage of alternations and **(C)** number of total entries in mTBI and SHAM groups, bars represent mean ± SEM. **p < 0.01. NOR test: **(D)** Discrimination index (DI > 0, preference for NOb; DI < 0, preference for FOb; DI = 0, null preference), bars represent median ± interquartile range and **(E)** total exploratory time; bars represent mean ± SEM. *p < 0.05. **(F)** Experimental design for fear conditioning after mTBI. **(G)** Percentage of freezing during Test 1 or Test 2 in conditioned (CA-US) and non-conditioned (CA-noUS) mTBI and SHAM groups, bars represent mean ± SEM. *p < 0.05, ***p < 0.001. SHAM, control group; mTBI, mild traumatic brain injury group; d, day; h, hour; SEM, standard error of the mean.

A total of 86 animals were used for the behavioral tests presented in Figure 1. Animals were randomly assigned to either the SHAM or mTBI group and subjected to the mTBI protocol described above. Independent subsets of animals were used for different behavioral tasks (Y-maze: *n* = 16 SHAM, 11 mTBI; Novel Object Recognition (NOR): *n* = 13 SHAM, 9 mTBI; and fear conditioning: *n* = 18 SHAM, 19 mTBI. No pharmacological treatment was administered to these animals (Figures 1A, G), but to reduce the number of animals used, animals from fear conditioning and Ymaze tests received two saline injections, and were the same used in group 2 as controls of treatments for this behavioral test.

#### 2.6.2 Group 2

**FIGURE 2:**
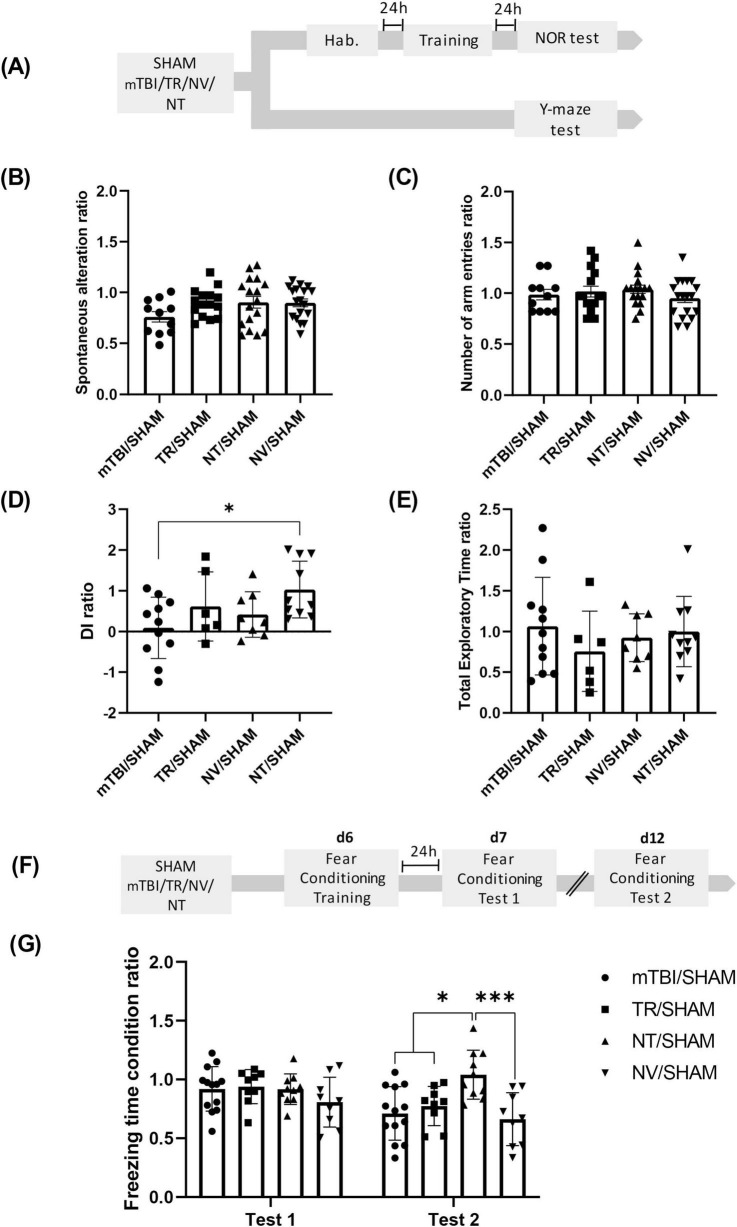
NT treatment mitigates cognitive deficits induced by mTBI **(A)** Schematic representation of the experimental design for the Y-maze and NOR tests following mTBI and treatment administration. Y-maze test: **(B)** Percentage of alternations ratio and **(C)** number of total arms entries ratio among experimental groups. NOR test: **(D)** Discrimination index ratio and **(E)** total exploration time ratio across groups. Bars represent mean ± SEM. *p < 0.05. **(F)** Schematic of the fear conditioning experimental timeline. **(G)** Percentage of freezing time ratio during Test 1 or Test 2 in conditioned (CA-US) and non-conditioned (CA-noUS) animals across mTBI and treatment groups. Bars represent mean ± SEM. *p < 0.05, ***p = 0.0003. SHAM, control group; mTBI, mild traumatic brain injury group; TR, triamcinolone acetonide; NV, blank lipid nanocapsules; NT, TR-loaded lipid nanocapsules; d, day; h, hour; ±SEM, standard error of the mean.

A total of 179 animals were included for the behavioral tests shown in Figure 2. Animals were randomly divided into SHAM and mTBI groups and underwent the mTBI protocol as previously described. Animals that receive the impact were randomly allocated into four treatment subgroups, based on the compound administered intraperitoneally (i.p.) 15 min (min) and 24 h (h) after mTBI: saline (mTBI group), NT at a dose equivalent to 0.5 mg/kg of TR (NT group), TR solution at 0.5 mg/kg (TR group), or NV (NV group). SHAM animals received two saline injections in volumes matched to the treatment groups, except for those in the fear conditioning task, where the same animals used in Group 1 (Shock SHAM and mTBI) were employed for comparison with TR, NT, and NV groups. Independent animal groups were used for each behavioral test (Y-maze: *n* = 16 SHAM, 11 mTBI, 18 NV, 15 TR, 17 NT; NOR: *n* = 11 SHAM, 12 mTBI, 9 NV, 7 TR, 10 NT; and fear conditioning: *n* = 12 SHAM, 13 mTBI, 9 NV, 9 TR, 10 NT) (see Figures 2A, F).

#### 2.6.3 Group 3

**FIGURE 3:**
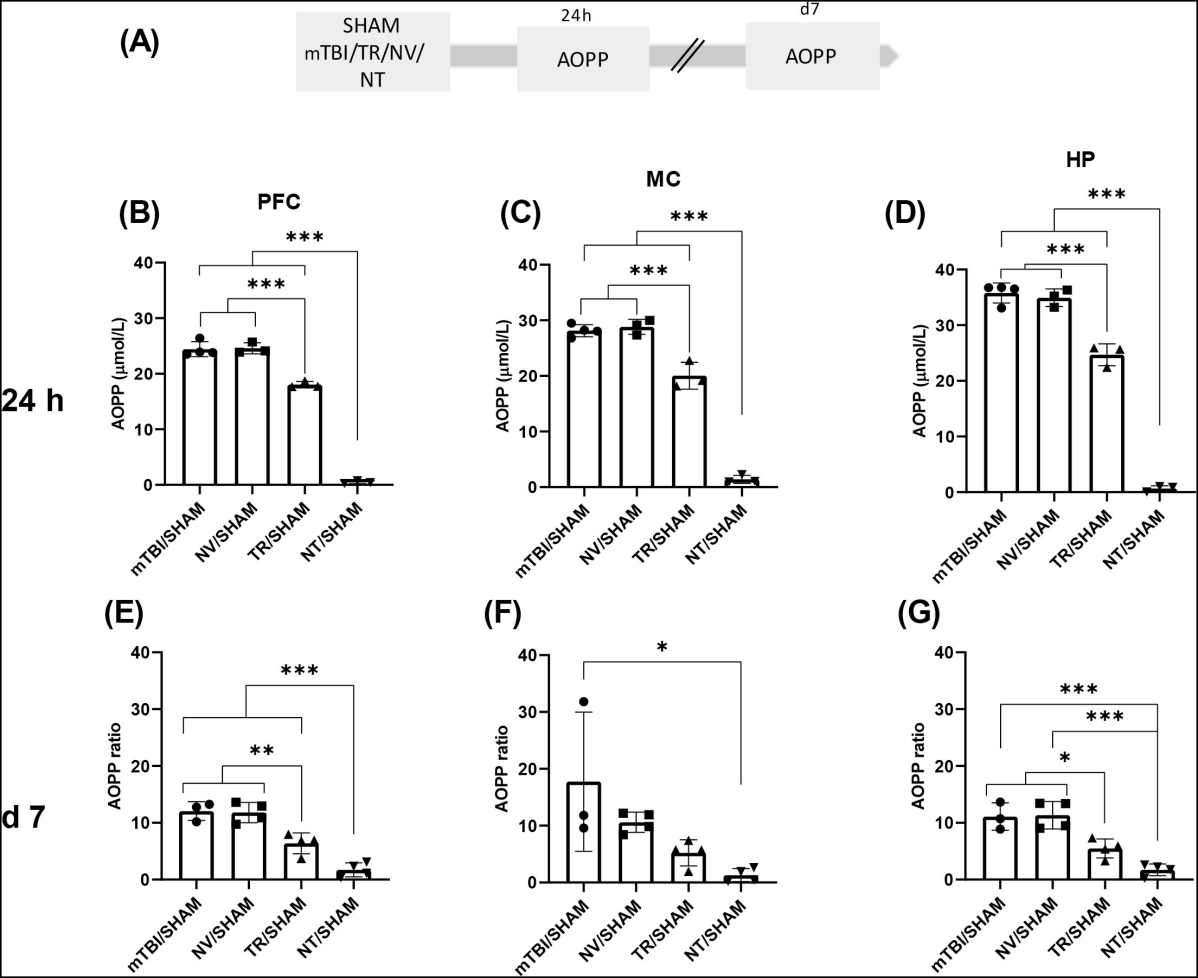
Early treatment with NT reduced protein peroxidation induced by mTBI **(A)** Schematic representation of the experimental design for AOPP quantification, a marker of protein peroxidation. AOPP ratio in distinct brain regions 24 h and 7 days after mTBI: **(B,E)** PFC, **(C,F)** MC, **(D,G)** HP. Bars represent the mean ± SEM. *p < 0.05, **p < 0.005, ***p < 0.0001. SHAM, control group; mTBI, mild traumatic brain injury group; TR, triamcinolone acetonide; NV, blank lipid nanocapsules; NT TR-loaded lipid nanocapsules; d, day; h, hour; ±SEM, standard error of the mean. AOPP (advanced oxidation protein products), OS (oxidative stress), PFC (prefrontal cortex), MC (motor cortex), HP (hippocampus).

**FIGURE 4:**
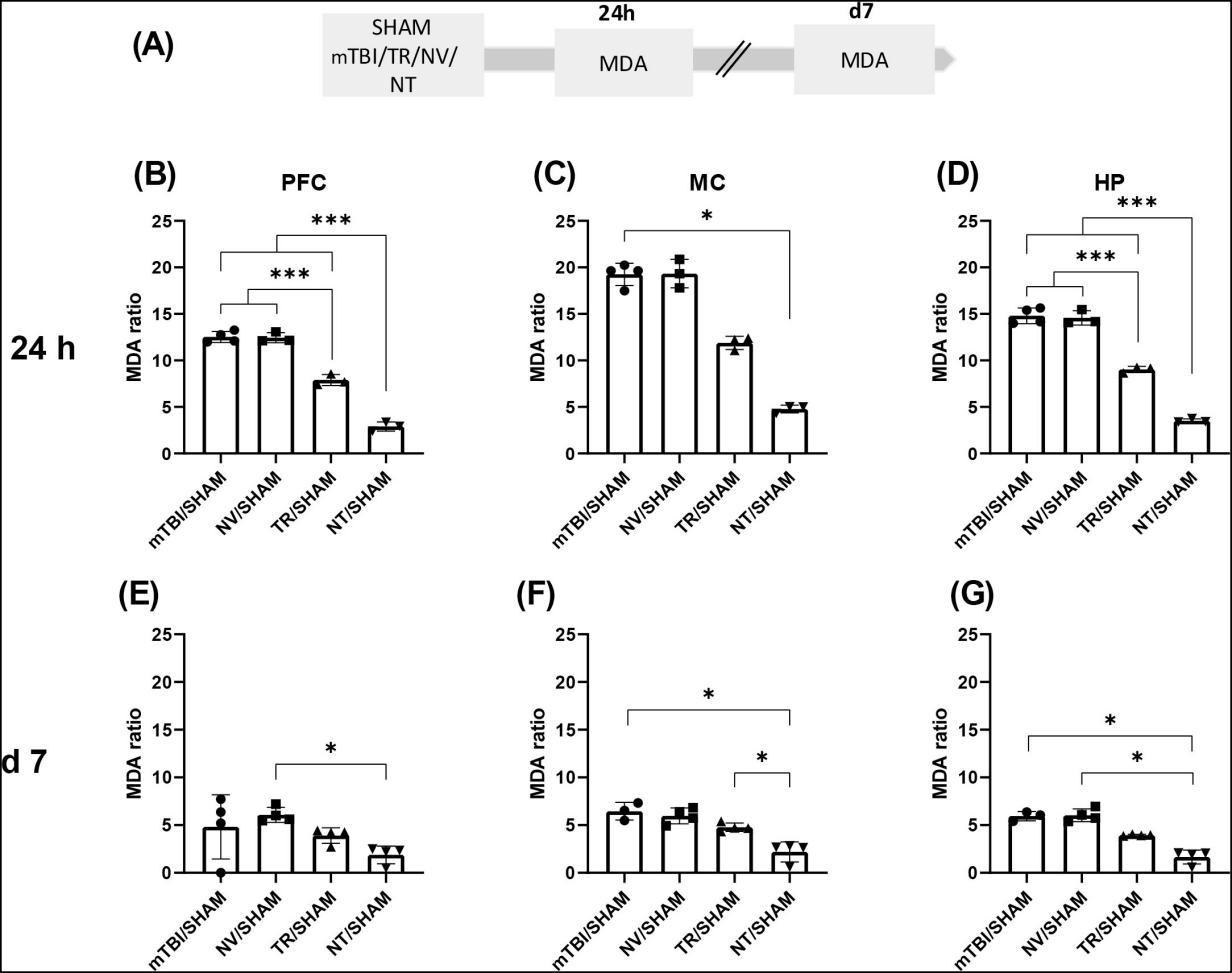
Early treatment with NT reduced lipid peroxidation induced by mTBI **(A)** Schematic representation of the experimental design for MDA quantification, a biomarker of lipid peroxidation. MDA ratio in different brain regions at 24 h and 7 days after mTBI: **(B,E)** PFC; **(C,F)** MC; **(G,D)** HP. Bars represent the mean ± SEM. *p < 0.05, ***p < 0.0001. SHAM, control group; mTBI, mild traumatic brain injury group; TR, triamcinolone acetonide; NV, blank lipid nanocapsules; NT, TR-loaded lipid nanocapsules; d, day; h, hour; ±SEM, standard error of the mean. AOPP (advanced oxidation protein products), OS (oxidative stress), PFC (prefrontal cortex), MC (motor cortex), HP (hippocampus).

30 animals were used to assess advanced oxidation protein products (AOPP) and malondialdehyde (MDA) levels. Animals were subjected to the mTBI protocol and randomly assigned to five experimental groups: SHAM, mTBI, NV, TR, and NT, receiving the same treatments as described for Group 2. Half of the animals were sacrificed 24 h after mTBI, and the remaining animals at 7 days post-injury (see Figures 3A, 4A). Each group consisted of three animals per time point.

#### 2.6.4 Group 4

**FIGURE 5:**
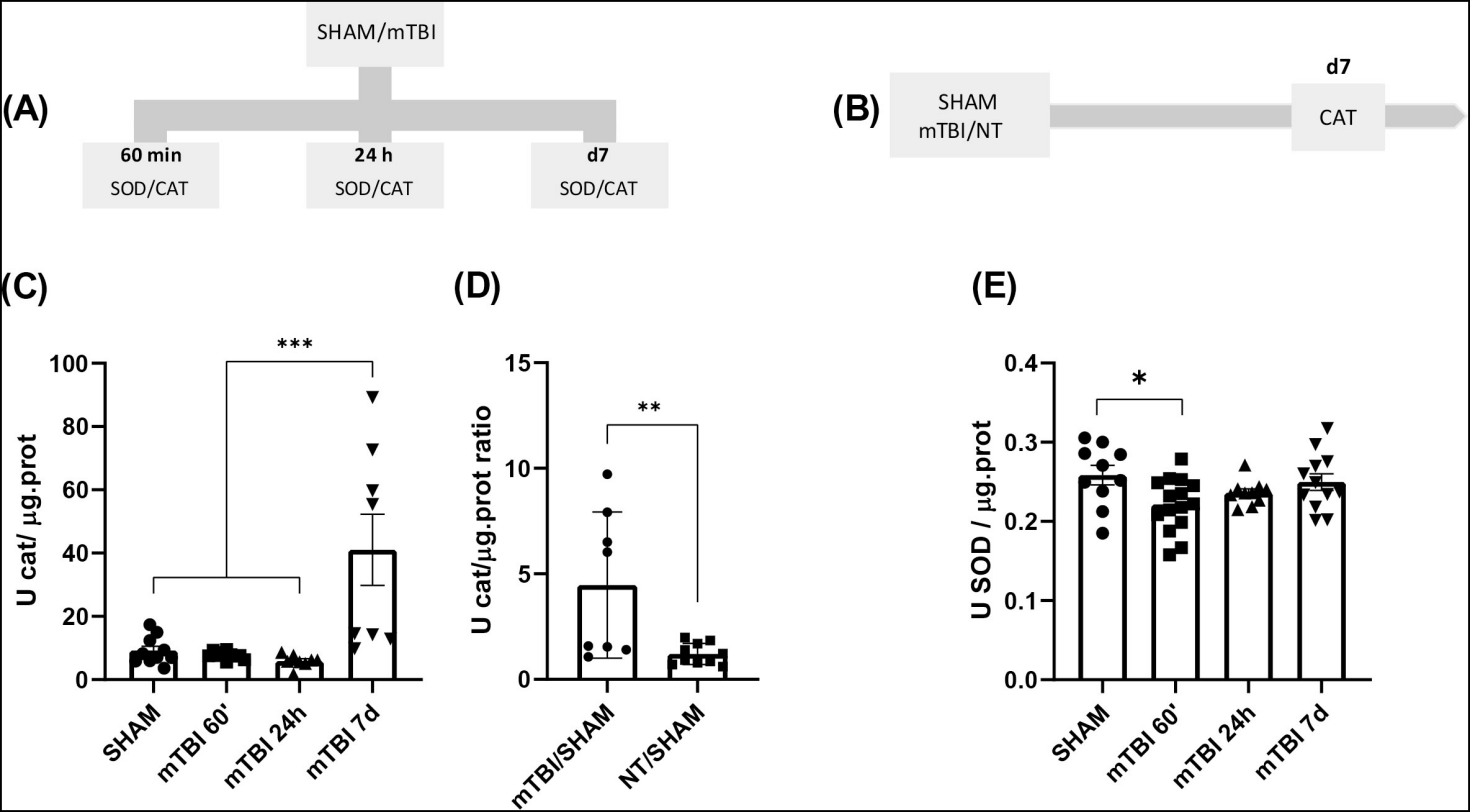
mTBI increases antioxidant enzymes activity and recovery after NT treatment **(A)** Schematic of the experimental timeline for the quantification of CAT and SOD activity in SHAM and mTBI groups. **(B)** Experimental design for CAT quantification following NT treatment. **(C,E)** Quantification of CAT and SOD activity, respectively, at 60 min, 24 h, and 7 days post-mTBI in SHAM and mTBI groups. **(D)** CAT activity ratio 7 days post-injury in TBI and NT groups. Bars represent the mean ± SEM. *p < 0.05, **p < 0.001, ***p < 0.0001. SHAM, control group; mTBI, mild traumatic brain injury group; NT, TR-loaded lipid nanocapsules; CAT, catalase; SOD, superoxide dismutase; min, minutes; h, hour; d, day; ±SEM, standard error of the mean.

85 animals were used to evaluate the temporal profile of catalase (CAT) and superoxide dismutase (SOD) activities, as shown in Figure 5. Animals were randomly divided into SHAM and mTBI groups, subjected to the mTBI protocol, and sacrificed at the time points indicated in Figure 5A. Independent cohorts of animals were used for each time point. For CAT: SHAM (*n* = 10), mTBI at 60 min (*n* = 10), 24 h (*n* = 8), and 7 days (*n* = 8); for SOD: SHAM (*n* = 10), mTBI at 60 min (*n* = 15), 24 h (*n* = 10), and 7 days (*n* = 12).

#### 2.6.5 Group 5

28 animals were used to investigate the effects of NT treatment on CAT activity (see Figure 5B). Animals were randomly assigned to three groups: SHAM, mTBI, and NT. Following the mTBI protocol, animals in the NT group received NT as described for Group 2, while the SHAM and mTBI groups received saline. All animals were sacrificed at 7 days post-injury.

#### 2.6.6 Drug solutions and dosing regimen selection

The concentration of TR in the NT solution was 0.25 mg/mL. The standard TR was obtained by dissolving a commercially available injectable suspension in 0.1 M phosphate-buffered saline (PBS) to achieve a concentration of 0.4 mg/mL. All solutions were sterilized through filtration using a 0.22 μm hydrophilic sterile filter (JetBioFil) under a laminar flow hood and kept at 4°C–8°C until use. The TR dose was selected based on studies in rats that evaluated GCS treatment in models of rheumatic diseases, which reported both benefits and complications. Additionally, the CRASH study on dexamethasone therapy in TBI highlighted significant adverse events in the treated group. The dosing regimen of two administrations of 0.5 mg/kg was determined to be safe, considering the equivalence in the doses of various GCS as reported elsewhere (Tonelli, 1966; Rowland et al., 1983; Larsson et al., 2004; Roberts et al., 2004; Earp et al., 2008; Du et al., 2016; Noyin et al., 2018). Furthermore, the dosing regimen, two administrations separated by 24 h, was chosen to replicate a sub-acute administration within a time window proximal to mTBI (60 min and 24 h), during which elevated levels of OS markers have been previously characterized in this model (Montivero et al., 2021b).

### 2.7 Behavioral tests

#### 2.7.1 Y-maze

The Y-maze task allows the assessment of working memory and relies on the rodents’ innate curiosity to explore unfamiliar or previously unvisited areas. Normal performance requires interaction across different brain regions, such as hippocampus (HP) and prefrontal cortex (PFC) (Kraeuter et al., 2019).

The Y-Maze apparatus is made of three arms (50 × 10 × 20 cm) of waterproof wood, elevated to a height of 50 cm above the floor. The test was conducted as previously described (Montivero et al., 2021b), seven days after mTBI protocol. Briefly, rats were placed at the end of one arm, facing the center of the maze, and were allowed to explore the arms freely for 8 min. An entry was defined as the placement of the four paws into the arm, and spontaneous alternations (triplet of consecutive entries to different arms) were counted. A rat with a normal working memory will remember the arms of the maze that it has already visited and will show a tendency to enter the less recently visited arm. To be included in statistical analysis, animals must fulfill the inclusion criteria, having at least five entries and/or less than 2 min without a new movement or entry. The percentage of alternations was calculated using Equation 1.

The total number of entries was also quantified and considered as an indicator of locomotor activity.

For this test, some animals were subjected to the mTBI protocol (mTBI or SHAM) and then returned to their home boxes. Seven days thereafter, working memory was evaluated (Figure 1A). Other groups of animals were also subjected to the mTBI protocol, and 15 min or 24 h after, they received one of the treatments described above. Seven days after working memory was tested (Figure 2A).


(1) $$Alternations\left(\%\right)=\left(\frac{N^{{}^{\circ}}\,o\,f\,A\,l\,t\,e\,r\,n\,a\,t\,i\,o\,n\,s}{T\,o\,t\,a\,l\,e\,n\,t\,r\,i\,e\,s\,t\,o\,a\,r\,m\,s-2}\right)*100$$

#### 2.7.2 Novel object recognition

The NOR evaluates hippocampal-dependent long-term recognition memory (Antunes and Biala, 2012). The protocol, displayed in Figure 1A, consisted of three trials (habituation, training, and test) separated by an inter-trial interval (ITI) of 24 h (Constantin et al., 2025). During habituation, rats were placed into a 60 × 60 × 60 cm box for 10 min. During training, animals explored two identical plastic objects (familiar objects-FOb) placed in the box for 5 min. In the test, one object was replaced by another of a different shape (novel object-NOb), and rats explored the box for 5 min. The role (familiar or novel) of objects was kept constant, and the relative position of the two objects was counterbalanced and randomly permuted for each experimental animal. Objects were cleansed with ethanol (70%) between animals and trials. The time spent exploring NOb and FOb was taken manually. The discrimination index (DI) to assess preference for NOb or FOb was calculated using Equation 2. For this test, some animals were subjected to the mTBI protocol (SHAM and mTBI) and then returned to their home boxes. Five days after they were subjected to the habituation session, 24 h after they were trained, and the next day they were tested (Figure 1A). Other groups of animals were also subjected to the mTBI protocol, and 15 min or 24 h after they received one of the treatments described above, and then they were subjected to the NOR protocol (Figure 2A).


(2) $$D\,I=\left(\frac{N\,O\,b-F\,O\,b}{N\,O\,b+F\,O\,b}\right)*100$$

#### 2.7.3 Contextual fear conditioning

Fear conditioning evaluates long-term classical (Pavlovian) aversive memory, predicting the aversive event by associating the context (the conditioning apparatus-CA) with the threat (Izquierdo et al., 2016). A previously described protocol with modifications was used (Constantin et al., 2025; Figures 1F, 2F). The CA consists of a 25 × 22 × 22 cm box with a floor made of a stainless-steel grid connected to a shock generator. During training, animals were left in the CA for 3 min for acclimation. Then, they received three-foot shocks (0.6 mA, 3 s each, 60 s interval), followed by an additional 50 s period (CA-US groups). For this test, some animals were subjected to the mTBI protocol (SHAM and mTBI) and returned to their home boxes. Six days thereafter, they were subjected to the training session, 24 h after they were re-exposed to the CA (Test 1), and 5 days later, they were re-exposed to CA (Test 2) (Figure 1F). Other groups of animals were also subjected to the mTBI protocol, and at 15 min or 24 h they received one of the treatments described above and then subjected to the contextual fear conditioning protocol (Figure 2F). Other rats were placed in the CA, but they did not receive foot shocks (CA-noUS group). Animals returned to their home cages and, 24 h after, they were placed in the CA for 5 min without foot shock delivery (Test 1). Then, animals returned to their home cages, and 5 days later, they were re-exposed to the CA for 5 min without foot shock delivery (Test 2). Freezing behavior–defined as the complete absence of body and head movement, except for those related to breathing–was measured during Test 1 and Test 2.

### 2.8 Biochemical determinations

#### 2.8.1 Advanced oxidation protein products (AOPP) and malondialdehyde (MDA) quantification

Animals were sacrificed by guillotine 24 h or 7 days post-injury, their brains were removed, and PFC, motor cortex (MC) and HP were dissected (Figures 3, 4A). Immediately, tissue samples were then homogenized with PBS 0.1M and centrifuged for 10 min at 4°C and 12.000 *g*. The supernatant was diluted 1/30 for HP and 1/20 for PFC and MC in PBS 0.1M (sample). AOPP and MDA determinations were made as previously described (Montivero et al., 2021b) following the protocol described elsewhere (Katerji et al., 2019). For AOPP determinations, the sample (200 μL), chloramine T (0–100 μmol/L, Sigma-Aldrich, Argentina) for calibration or PBS 0.1 M pH 7 as blank, were added in different microtiter plate wells. Then, 10 μL of 1.16 M potassium iodide (Cicarelli, Argentina) and 20 μL of acetic acid (glacial, Cicarelli, Argentina) were added to each well, and absorbance was immediately read at 340 nm. The AOPP concentration was expressed as equivalent of chloramine units (μmol/L) per milligram of protein. For MDA determinations, 250 μL of trichloroacetic acid (Cicarelli, Argentina) and 250 μL of thiobarbituric acid (Merck, Argentina) were added to 500 μL of the diluted sample. Immediately after, samples were kept in boiling water for 10 min. Then, they were centrifuged at 1,000 rpm for 10 min, and after cooling to clear the supernatant from denaturalized proteins, absorbance was measured at 532 nm. Thiobarbituric acid-reactive substances were quantified using an extinction coefficient of 1.56 × 105 M^1^ cm^1^ and expressed as nmol of MDA per milligram of protein. In both AOPP and MDA determinations, initial tissue protein content was determined by using the Bradford reagent. Both biochemical determinations were performed in the same animal, immediately after sacrifice, using fresh brain tissue.

#### 2.8.2 Catalase and superoxide dismutase enzymatic activity

Animals were sacrificed by guillotine and their brains were removed. Brain areas related to the performance in the behavioral test were dissected (PFC, MC, dorsal HP and basolateral amygdala), immediately frozen and stored at −80°C until further analysis (Figures 5A, B). Due to the limited amount of tissue available from each brain area per animal, dissected areas were pooled for each animal. Briefly, tissue samples were homogenized in PBS buffer pH 6.25; thereafter, the homogenate was centrifuged at 10000 rpm and 4 °C for 30 min. The supernatant (sample) was collected and used for measurements.

Catalase enzymatic activity was measured by its ability to decompose hydrogen peroxide (H2O2) into water (H2O) and oxygen (O2). The enzyme’s activity was determined by the Aebi method which involves monitoring the disappearance of H2O2 at 240 nm (Santos et al., 2005). In a cuvette with 2.5 mL of isotonic sodium phosphate buffer pH 7.0, 40 μL of sample and 200 μL of hydrogen peroxide (150 mM, Cicarelli, Argentina) were added. After homogenization, the initial absorbance was measured at 240 nm, and the measurement was repeated after 5 min. CAT activity was expressed as the change in absorbance per μg of protein in the homogenate.

Superoxide dismutase activity is measured based on its ability to inhibit the photochemical reduction of nitro blue tetrazolium (NBT). Light activates riboflavin, which becomes excited and loses an electron, reducing oxygen to a superoxide ion. The superoxide ion then loses an electron, with methionine acting as a catalyst, reducing NBT to blue formazan. In the presence of SOD, the increase in NBT color is diminished because the enzyme dismutates the superoxide ion. Consequently, less NBT is reduced to blue formazan, resulting in a decreased optical density measured at 560 nm. Briefly, 100 μL of sample was added to Khan tubes together with 100 μL of NBT (Sigma, Argentina), 300 μL of methionine (Sigma, Argentina), 300 μL of EDTA (Sigma, Argentina), and 300 μL of riboflavin (Sigma, Argentina). Duplicates of samples were measured, and two blanks were run using 100 μL of water instead of the sample. One blank (dark control) was incubated in the dark for 45 min, while the second blank (light control) and the samples were incubated under light for 45 min. Afterward, 200 μL from each tube was transferred to a well of a 96-well microplate. Absorbance was measured for SOD activity quantification.

### 2.9 Statistical analyses

Normal data distribution was confirmed by Shapiro-Wilk test. Two-tailed Student’s *t*-test was used for Y-maze, NOR and CAT activity under NT treatment results. One-way ANOVA, with Tukey’s multiple comparison as a *post-hoc* test was used for Y-Maze, NOR in different treatments groups, AOPP and MDA at 24 h, and CAT and SOD activities without treatments. Results from fear conditioning were analyzed by two-way ANOVA with repeated measurements in one factor, followed by Bonferroni’s multiple comparison test as *post-hoc*. On the other hand, data with non-normal distribution were analyzed by using Mann-Whitney test for two groups comparisons (NOR DI), or Kruskal-Wallis (MDA 24 h, 7 days) followed by Dunn’s *post-hoc* test, for more than two groups comparisons. A 95% confidence level was considered for all analysis. For behavioral and biochemical experiments in groups with pharmacological treatments, individual values were divided by the average of each parameter obtained in the SHAM group. The ratios between the values obtained for each animal were then used for statistical comparisons, and graphs that are presented in Figures 2–4. The effects of each treatment in mTBI animals related to SHAM (NT/SHAM, NV/SHAM, and TR/SHAM) were then compared with the control group (mTBI/SHAM), considering them as independent groups and analyzed by using one-way (Y-Maze, NOR, AOPP and MDA) or two-way ANOVA (fear conditioning) to reduce the number of animals used.

## 3 Results

### 3.1 mTBI induced deficits in working memory, novel object recognition, and fear conditioning

Working memory is one of the cognitive domains most vulnerable to mTBI and has been shown to predict patient outcomes (Ricker et al., 2001; Wylie et al., 2015). In the present study, we replicated previous findings from our laboratory (Montivero et al., 2021b) by applying an identical mTBI protocol, and testing working memory 7 days thereafter (Figure 1A). A significant reduction in spontaneous alternation in the Y-maze was observed in the mTBI group compared to the SHAM controls [*t* = 3.246; df = 25; *p* = 0.033] (Figure 1B), indicating impaired working memory. Importantly, no significant differences were found in the total number of arm entries between groups [*t* = 0.8457; df = 25; *p* = 0.1966] (Figure 1C), suggesting that the decreased spontaneous alternation was not due to alterations in locomotor activity.

The NOR test is a well-established paradigm to assess recognition memory, which is notably dependent on hippocampal function during the consolidation phase (Karve et al., 2016). To determine the impact of mTBI on recognition memory, SHAM and mTBI groups were compared (Figure 1A). As shown in Figure 1D, the DI was significantly lower in the mTBI group relative to SHAM controls [*U* = 31; *p* = 0.0341], indicating impaired recognition memory. Total exploration time of both objects did not differ significantly between groups [*t* = 1.395, df = 20; *p* = 0.1784] (Figure 1E), confirming that the reduction in NOb exploration was not attributable to decreased locomotion.

For the behavioral analysis during the Test 1 and Test 2, used as repeated factor, a repeated measures ANOVA for the percentage of time spent freezing revealed a significant effect of the condition (SHAM or mTBI CA-noUS and SHAM or mTBI CA-US) [F (3, 33) = 26.37; *p* < 0.0001]; period of evaluation (R1 factor, test 1 and test 2) [F (1, 33) = 36.30; *p* < 0.0001] and period of evaluation x conditioning [F (3, 33) = 8.035; *p* = 0.0004] (Figure 1G).

From the relevant statistical information, the Bonferroni’s multiple comparisons test revealed that, during test 1, no differences were found between SHAM CA-US vs. mTBI CA-US (*p* = 0.3492), but both groups were different from their respective CA-noUS (*p* < 0.0001). Interestingly, during test 2 mTBI CA-US group showed significantly lower % of freezing than the SHAM CA-US group (*p* = 0.0009), without differences with the CA-noUS groups. Although, the freezing values in the SHAM CA-US group in test 2 were significantly lower vs. the group values during test 1 (*p* < 0.001), this group presented higher freezing response in comparison to SHAM and mTBI CA-noUS groups (*p* < 0.0001). Taken together these results indicate that mTBI did not affect fear memory consolidation (test 1), but it impaired memory retention (test 2).

### 3.2 Early treatment with NT mitigated the expression of cognitive deficits in animals with mTBI

The nanoformulations used for treatment (NT and NV) exhibited colloidal characteristics consistent with previously reported data (Formica et al., 2020). Specifically, NV displayed an APS of 46.83 ± 0.92 nm, a PDI of 0.092, and a zeta potential of −19 ± 2 mV. Similarly, NT showed an APS of 43.71 ± 1.18 nm, a narrow size distribution (PDI = 0.083), and a zeta potential of −13 ± 1 mV.

To assess whether early NT therapy prevents cognitive deficits induced by mTBI, we performed a comprehensive behavioral evaluation using the Y-maze, NOR, and fear conditioning paradigms in animals from Group 2, as described in the “Section 2.6 Experimental Groups and Drug Treatment” (Figures 2A, F). In the Y-maze test, no significant differences were found in the spontaneous alternation ratio among groups [F (3, 57) = 1.759; *p* = 0.1653], indicating that neither NT nor control treatments restore working memory deficits (Figure 2B). Additionally, any treatment affected general locomotor activity, as reflected by the comparable number of arm entries across groups [F (3, 57) = 0.7131; *p* = 0.5482] (Figure 2C).

Conversely, NOR test results exhibited a significant main effect of treatment on recognition memory, as shown by differences in the DI among groups [F (3, 31) = 3.119; *p* = 0.0401] (Figure 2D). *Post-hoc* analysis revealed that the NT/SHAM group exhibited a significantly higher DI compared to the mTBI/SHAM group (*p* = 0.0250) without differences detected between other groups (*p* > 0.05). These findings suggest a protective effect of NT therapy against mTBI-induced recognition memory impairments. Furthermore, total exploration times did not differ among groups [F (3, 31) = 0.5792; *p* = 0.6331] (Figure 2E), confirming that changes in DI were not attributable to altered exploratory behavior.

For fear conditioning analysis during the Test 1 and Test 2, used as repeated factor, the two-way ANOVA with repeated measures in the time factor revealed a significant effect of treatment (mTBI/SHAM, TR/SHAM, NT/SHAM and NV/SHAM) [F (3, 37) = 3.575; *p* = 0.0229], period of evaluation (R1 factor, Test 1 and Test 2) [F (1, 37) = 10.80; *p* = 0.0022] and the interaction period of evaluation × treatment [F (3, 37) = 6.53; *p* = 0.0012] (Figure 2G).

Consistent with SHAM and mTBI groups during Test 1, all treated groups showed freezing behavior comparable to mTBI/SHAM controls, indicating that treatments maintain intact fear memory acquisition and expression. However, during the retention test (Test 2), the *post-hoc* analysis revealed that the NT/SHAM group exhibited significantly higher freezing percentages compared to both mTBI/SHAM (*p* = 0.0007), NV/SHAM (*p* = 0.0003) and TR/SHAM (*p* = 0.0211) groups. No differences were found between mTBI/SHAM, NV/SHAM and TR/SHAM (*p* > 0.05). This indicates that NT treatment conferred protection against mTBI-induced long-term fear memory deficits.

In Supplementary Figure 1, individual results for all parameters quantified in each behavioral test were expressed as the average of the row data obtained for each animal in each experimental group, to present a direct comparison between SHAM and mTBI animals, but those date were not statistically analyzed (Y-maze Supplementary Figures 1B, C; NOR Supplementary Figures 1D, E; fear conditioning Supplementary Figures 1G, H).

Taken together, these results suggest that early administration of NT effectively prevents recognition and long-term memory impairments associated with mTBI, although its efficacy in restoring working memory appears limited.

### 3.3 Early treatment with NT prevented increments in OS markers at 24 h and 7 days post-mTBI

Previous findings from our laboratory described early increases in OS markers after mTBI, including AOPP and MDA, which appeared at 60 min and peaked at 24 h in HP, PFC, and MC. These elevated levels persisted up to 7 days post-injury compared to SHAM (Montivero et al., 2021b). In the present study, those findings were replicated in our animal model, and the efficacy of early NT administration in modulating these markers was evaluated. Supplementary Figures 2, 3 showed bar graphs performed with row data from AOPP and MDA determinations obtained in animals described as Group 3 in “Section 2 Materials and Methods,” to allow a direct comparison between SHAM and mTBI animals.

Advanced oxidation protein products and MDA concentrations were measured at 24 h or 7 days post-injury in brain areas from animals receiving pharmacological treatments (NV, NT, or TR) or saline (SHAM and mTBI) (Figures 3, 4A). Individual values of each animal were normalized to the mean of the SHAM group and expressed as “ratio.” At 24 h, a significant main effect of treatment was detected on AOPP levels across all brain regions–MC [F (3, 9) = 230.7; *p* < 0.0001], PFC [F (3, 9) = 439.1; *p* < 0.0001], and HP [F (3, 9) = 331.3; *p* < 0.0001] (Figures 3B–D). *Post-hoc* analyses revealed that both TR/SHAM (MC *p* = 0.0003; PFC and HP *p* < 0.0001) and NT/SHAM (MC; PFC and HP (*p* < 0.0001) groups exhibited significantly lower AOPP levels than the untreated mTBI/SHAM group. Notably, AOPP levels in the NT/SHAM group were significantly lower than those in the TR/SHAM group, indicating superior efficacy of NT treatment in mitigating the early AOPP surge in MC (mean difference: 18.63; *p* < 0.0001), PFC (mean difference: 17.58; *p* < 0.0001), and HP (mean difference: 24.03; *p* < 0.0001). Importantly, the mean ratio of AOPP levels between NT and SHAM groups was 0.65 ± 0.3, suggesting near normalization to SHAM levels.

Similarly, MDA levels demonstrated a significant treatment effect at 24 h in MC [*U* = 9.89; *p* = 0.0014, Kruskal-Wallis with Dunn’s *post-hoc* test], PFC [F (3, 9) = 213.7; *p* < 0.0001], and HP [F (3, 9) = 227.6; *p* < 0.0001] (Figures 4B–D). Lipid peroxidation was significantly reduced in corticosteroid-treated groups in PFC and HP (NT/SHAM and TR/SHAM) compared to mTBI/SHAM and NV/SHAM (*p* < 0.0001). Moreover, MDA levels in NT/SHAM from all brain structures were significantly lower than in TR/SHAM (*p* < 0.0001), further demonstrating the enhanced efficacy of NT relative to TR at equivalent doses.

Seven days post-mTBI, different groups of animals were similarly treated and sacrificed, with AOPP and MDA levels assessed in the same brain regions (Figures 3E–G, 4E–G). A significant effect of treatment on AOPP levels was observed in MC [F (3, 11) = 5,821; *p* = 0,0124], PFC [F (3, 11) = 33.89; *p* < 0.0001], and HP [F (3, 11) = 22.26; *p* < 0.0001] (Figures 3E–G). In MC, *post-hoc* testing revealed significantly lower AOPP levels in the NT/SHAM group compared to mTBI/SHAM (*p* = 0,0106), with no significant differences among other groups. In PFC, both corticosteroid treatments (NT/SHAM and TR/SHAM) significantly reduced AOPP levels compared to mTBI/SHAM (*p* < 0.0001 and *p* = 0.004, respectively) and NV/SHAM (*p* < 0.0001 and *p* = 0.0032, respectively), with NT/SHAM levels significantly lower than TR/SHAM (*p* = 0.0096), indicating greater efficacy of NT. Similar trends were observed in HP.

Malondialdehyde levels at 7 days exhibited patterns comparable to AOPP across analyzed regions: MC [*U* = 11.74; *p* = 0.0002], PFC [*U* = 8.316; *p* = 0.0233], and HP [*U* = 12.1; *p* < 0.0001] (Figures 4E–G). Dunn’s multiple comparisons test indicated that MDA values in the NT/SHAM group were significantly lower than those in mTBI/SHAM (MC *p* = 0.0175; HP *p* = 0.0325) or NV/SHAM (MC *p* = 0.0339; PFC *p* = 0.0360; HP *p* = 0.0160) groups.

In Supplementary Figures 2, 3, individual results for AOPP and MDA determinations at 24 h or 7 days in all brain structures were expressed as the average of the row data obtained for each animal in each experimental group, to present a direct comparison between SHAM and mTBI animals, but those data were not statistically analyzed.

### 3.4 mTBI altered antioxidant activity markers and its prevention with early treatment with NT

Based on the results obtained in the previous section, we investigated whether mTBI affects the antioxidant system by altering the activity of enzymes that serve as the first line of antioxidant defense, such as CAT and SOD, in brain regions associated with the behavioral outcomes observed in this study. These experiments were performed in animals from Group 4, described in “Section 2 Material and Methods” (Figure 5A). Additionally, we assessed whether NT treatment could restore their activity in Group 5. Figures 5B, C illustrates the time-dependent profile of CAT activity, revealing a main effect of condition (mTBI vs. SHAM) [F (3, 32) = 10.19; *p* < 0.0001]. The post-hoc indicated a significant increase on day seven post-mTBI compared to other time points (*p* = 0,0005). Furthermore, early NT treatment prevented the rise in CAT activity on day 7 post-mTBI, as evidenced by a statistically significant reduction in the CAT activity ratio for mTBI-NT/SHAM compared to mTBI/SHAM (*t* = 2.972, df = 16; *p* = 0,0090) (Figure 5D). No significant differences were observed among other groups relative to mTBI/SHAM (*p* > 0.05).

Conversely, following mTBI the activity pattern over time of SOD was evaluated. A significant main effect of condition (mTBI vs. SHAM) was found in Figure 5E [F (3, 43) = 3.073; *p* = 0.0376]. Specifically, mTBI caused a significant decrease in SOD activity at 60 min post-injury (*p* = 0.0392) compared to SHAM, while no differences were observed at 24 h or 7 days post-injury (*p* > 0.05).

## 4 Discussion

The present investigation further characterizes the cognitive and emotional sequelae induced by mTBI in closed-head animal model. Our data confirm that mTBI causes significant impairment in recognition memory as well as in fear conditioning seven days after injury. These deficits span multiple cognitive domains and serve as preclinical manifestations of PCS. Furthermore, it revealed for the first time the effectiveness of NT in preventing many cognitive deficits induced by mTBI. The i.p. administration of NT demonstrated much more effectiveness than TR in its commercial formulation in preventing both OS increments and behavioral outcomes in NOR and fear conditioning.

In line with recent findings in animal models and humans (Akcay et al., 2025; Exline et al., 2025; Lawton et al., 2025), previous reports from our laboratory have demonstrated that mTBI, using the same settings in the weight-drop model of TBI, induces persistent cognitive impairments, specifically deficits in working memory. This was accompanied by a rapid and sustained increase in OS markers, such as AOPP and MDA. These markers remained elevated for up to 7 days post-injury, indicating ongoing secondary injury processes involving oxidative damage (Montivero et al., 2021b). In the present investigation, we reproduced those results (See Supplementary Figures 2, 3), and these findings align with numerous studies that have documented increased lipid peroxidation and protein oxidative damage in TBI models (Hall et al., 2010; Mohammadzadeh et al., 2018; Altinoz et al., 2016; Salem et al., 2022) as well as in brain tissues from TBI patients (Cristofori et al., 2001). The reversible and irreversible alterations of those macromolecules predispose individuals to a wide range of disorders, including neurodegenerative diseases (Poprac et al., 2017; Dröge, 2002), post-traumatic stress disorder (Prasad and Bondy, 2015), among others. These impairments may arise from the early activation of neuroinflammatory cascades following secondary injury mechanisms that disrupt critical neuronal networks required for memory processing (Babior, 2000; Block et al., 2006; Block and Hong, 2007). An increase in OS markers, such as AOPP and MDA, in both the hippocampus and prefrontal cortex supports this notion (Montivero et al., 2021b), as OS can disrupt neuronal excitability and synaptic plasticity, further contributing to cognitive impairment. Extending these observations, the present study provides, to the best of our knowledge, the first evidence of such biomarkers elevations using a weight-drop model involving a 45 g stainless steel ball dropped from 1 meter, without craniotomy, to induce closed-head mTBI in rats. Previous studies utilizing this model in rodents have predominantly employed heavier projectiles ranging from 200 to 400 g or more (Bodnar et al., 2019). While this methodological difference may represent a limitation in terms of standardization, it also offers a distinct advantage: most animals subjected to this protocol survive the injury and retain essential physiological functions such as feeding and hydration. This outcome closely parallels clinical cases of mTBI, in which patients are discharged following recovery with normal GCS scores. Furthermore, the specific weight and height parameters used in our model have been previously validated in other experimental paradigms (Saxena et al., 2024; Lirio et al., 2024; Sarı et al., 2021), supporting their relevance and translational value.

Furthermore, our study addresses emotional disturbances frequently associated with mTBI, a topic of increasing clinical relevance given the high prevalence of anxiety and affective disorders reported in patients with a history of mTBI (Sanders et al., 2025). There is also a strong epidemiological and mechanistic link between mTBI and post-traumatic stress disorder (PTSD), involving traumatic memory dysfunctions (Kennedy et al., 2007; Tanielian, 2008; Brenner et al., 2017). Consistent with these observations, we found that animals with mTBI exhibited reduced long-term retention of fear memory 12 days post-injury, without affecting acquisition or consolidation of fear memory. These findings align with previous reports of impaired fear memory recall in mouse models combining TBI and PTSD-like features (Teutsch et al., 2018) and contextual fear memory deficits in controlled cortical impact TBI models (Celorrio and Friess, 2023). This dissociation suggests that mTBI disrupts the consolidation or retrieval of emotionally salient memories rather than the initial learning process. This disruption may occur through neuroinflammatory and oxidative pathways that affect the amygdala-hippocampus-prefrontal circuits involved in emotional regulation (Kovesdi et al., 2012; Krukowski et al., 2021; Adkins et al., 2023).

The study’s novel aspect is the proposal and evaluation of a new pharmacological strategy to prevent cognitive and emotional sequelae induced by mTBI, through the repositioning of TR loaded into a lipid-based nanocarrier (NT). This formulation leverages TR’s mechanism of action–limiting arachidonic acid release and downstream neuroinflammatory mediator synthesis, the first step in the OS pathways (Ismail et al., 2020). It also enhances CNS access via a cutting-edge nanotechnology-based delivery system (Ismail et al., 2020; Groo et al., 2015). NT was more effective than conventional TR at preventing recognition memory and fear retention deficits, when administered early post-injury. Although NT did not fully reverse working memory deficits under the experimental conditions of the study, its selective protection of the circuits responsible for hippocampal-dependent memory and emotional processing represents a significant advancement in mTBI therapeutics. Conventional TR at equivalent dose was less effective, highlighting the critical role of nanocarrier-mediated delivery in overcoming the BBB and achieving therapeutic CNS concentrations.

The beneficial effects of NT treatment are likely mediated by targeting OS, as mTBI induced significant increases in AOPP and MDA in key brain regions, reflecting heightened oxidative damage. Early NT administration prevented these increases, reducing acutely (within 24 h) and maintaining OS markers at levels comparable to those of the SHAM at later stages (after 7 days). This pharmacological target has been proposed by other therapeutic options for TBI (Ismail et al., 2020). Furthermore, NT treatment reduced OS markers more effectively than TR treatment, highlighting the enhanced CNS delivery conferred by the nanocarrier system.

Together with SOD and the glutathione system, CAT constitutes a cellular defense mechanism against OS (see Halliwell and Gutteridge, 1989; Sies, 1991). CAT also plays a role in brain metabolism, including the metabolism of ethanol (Aragon and Amit, 1992; Correa et al., 2001). Additionally, mammalian CAT exhibits oxidase activity that is distinct from its recognized catalytic and peroxidic activities (Chelikani et al., 2004; Chance et al., 1952). Those activities are inactivated at high hydrogen peroxide concentrations, whereas oxidase activity is readily saturable and reversible (Vetrano et al., 2005). Besides, CAT expression or activity in brain has been increased after d-amphetamine administration (Carvalho et al., 2001) or lead exposure (Mattalloni et al., 2013). Accordingly, in the present investigation, we found significant increases in CAT activity at seven days after mTBI, while SOD activity was increased only at 24 h after mTBI and spontaneously returned to SHAM values on day 7. This induction of antioxidant activity could be attributed to a compensatory response to the increases in OS levels. Although, we cannot discharge that some of the OS can be induced by CAT oxidase activity. Accordingly, prevention of AOPP and MDA increased levels by NT treatment, could also prevent the increased CAT activity observed at 7 days after mTBI, normalize it and further prevent a possible CAT oxidase activity. By restoring this antioxidant capacity, NT likely mitigates reactive oxygen species (ROS)-mediated neuronal injury and dysfunction.

Given the established interplay between neuroinflammation and OS, wherein inflammatory processes amplify ROS production and contribute to neuronal damage, the ability of NT to modulate both pathways is particularly relevant. This biochemical stabilization may help preserve neuronal excitability and synaptic function within the HP, PFC, and associated networks, thereby supporting the observed preservation of cognitive and emotional behaviors.

Despite these promising findings, several limitations should be acknowledged. Although the efficacy of NT was indirectly supported by behavioral and biochemical outcomes, the lack of direct biodistribution and pharmacokinetic studies–unlike what has been demonstrated for other compounds (Ayoub et al., 2024)–limits the confirmation of enhanced brain delivery in our model. Furthermore, we did not assess additional neuroinflammatory parameters such as cytokine levels (e.g., IL-1β, IL-6, TNF-α) or glial activation markers (e.g., GFAP, S100β), which are essential to fully characterize the post-injury pathophysiology and the scope of NT’s therapeutic effects. The use of a 45 g free-fall weight-drop model without craniotomy, while reflecting key features of closed-head mTBI, is not yet widely standardized in the field, which may reduce comparability with more established preclinical models. Moreover, although NT was effective in the context of mTBI, future studies in moderate and severe injury models are warranted to determine its broader translational potential.

Additionally, it cannot be excluded that NT may exert greater therapeutic benefit when used in combination with other pharmacological or rehabilitative strategies. Investigating such multimodal approaches may contribute to a more comprehensive treatment framework that better addresses the multifactorial nature of TBI and minimizes long-term sequelae in affected individuals.

In conclusion, this study supports the repositioning of TR as a promising therapeutic agent for preventing long-term cognitive and emotional deficits following mTBI, by leveraging nanotechnology to enhance CNS delivery and efficacy. NT demonstrated significant effects in attenuating OS, restoring antioxidant capacity, and preserving cognitive and emotional functions. These findings open new avenues for pharmacological interventions in TBI with the potential to improve clinical outcomes. Nonetheless, further investigation is needed to confirm NT brain bioavailability, elucidate its effects on neuroinflammatory pathways, and validate its efficacy in more standardized and severe injury models. Moreover, incorporating NT into multimodal therapeutic strategies may offer a more integrative approach to addressing the diverse and complex sequelae of TBI across varying levels of severity.

## Data Availability

The raw data supporting the conclusions of this article will be made available by the authors, without undue reservation.

## Supplementary material

Supplementary Figure 1 NT treatment mitigates cognitive deficits induced by mTBI **(A)** Schematic representation of the experimental design for the Y-maze and NOR tests following mTBI and treatment administration. Y-maze test: **(B)** Percentage of alternations and **(C)** number of total arms entries among experimental groups. NOR test: **(D)** Discrimination index (DI > 0, preference for NOb; DI < 0, preference for FOb; DI = 0, null preference) and **(E)** total exploration time across groups. Bars represent mean ± SEM. **(F)** Schematic of the fear conditioning experimental timeline. **(G)** Percentage of freezing time during Test 1 or Test 2 in conditioned (CA-US) and non-conditioned (CA-noUS) animals across different mTBI and treatment groups. Bars represent mean ± SEM. SHAM, control group; mTBI, mild traumatic brain injury group; TR, triamcinolone acetonide; NV, blank lipid nanocapsules; NT, TR-loaded lipid nanocapsules; d, day; h, hour; ±SEM, standard error of the mean.

Supplementary Figure 2 Early treatment with NT reduced protein peroxidation induced by mTBI **(A)** Schematic representation of the experimental design for the quantification of AOPP, a marker of protein peroxidation. AOPP levels in distinct brain regions 24 h or 7 days after mTBI: **(B,E)** PFC, **(C,F)** MC, **(D,G)** HP. Bars represent the mean ± SEM. SHAM, control group; mTBI, mild traumatic brain injury group; TR, triamcinolone acetonide; NV, blank lipid nanocapsules; NT, TR-loaded lipid nanocapsules; d, day; h, hour; ±SEM, standard error of the mean. AOPP (advanced oxidation protein products), OS (oxidative stress), PFC (prefrontal cortex), MC (motor cortex), HP (hippocampus).

Supplementary Figure 3 Early treatment with NT reduced lipid peroxidation induced by mTBI **(A)** Schematic representation of the experimental design for the quantification of MDA, a biomarker of lipid peroxidation. MDA levels in different brain regions at 24 h or 7 days after mTBI: **(B,E)** PFC, **(C,F)** MC, **(D,G)** HP. Bars represent the mean ± SEM. SHAM, control group; mTBI, mild traumatic brain injury group; TR, triamcinolone acetonide; NV, blank lipid nanocapsules; NT, TR-loaded lipid nanocapsules; d, day; h, hour; ±SEM, standard error of the mean. AOPP (advanced oxidation protein products), OS (oxidative stress), PFC (prefrontal cortex), MC (motor cortex), HP (hippocampus).
