# Supplementary figures and images for: Triamcinolone-loaded nanocarriers: a novel strategy to mitigate cognitive and emotional sequelae induced by traumatic brain injury via modulation of oxidative stress
Source: Front Behav Neurosci. 2025 Aug 12;19:1638417. doi: 10.3389/fnbeh.2025.1638417 (PMC12378785; doi:10.3389/fnbeh.2025.1638417)

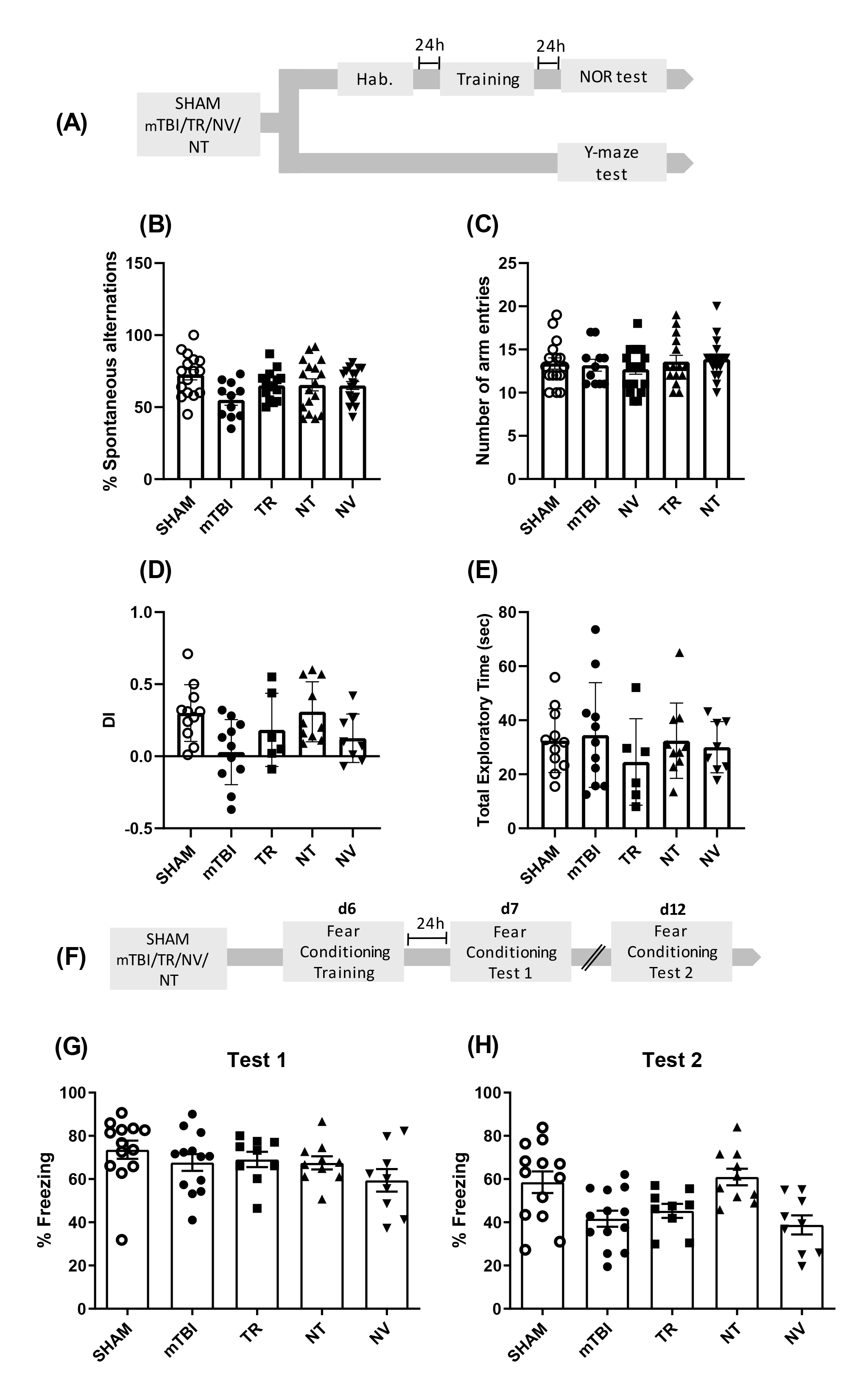

Supplement: Supplementary Figure 1 — NT treatment mitigates cognitive deficits induced by mTBI (A) Schematic representation of the experimental design for the Y-maze and NOR tests following mTBI and treatment administration. Y-maze test: (B) Percentage of alternations and (C) number of total arms entries among experimental groups. NOR test: (D) Discrimination index (DI > 0, preference for NOb; DI < 0, preference for FOb; DI = 0, null preference) and (E) total exploration time across groups. Bars represent mean ± SEM. (F) Schematic of the fear conditioning experimental timeline. (G) Percentage of freezing time during Test 1 or Test 2 in conditioned (CA-US) and non-conditioned (CA-noUS) animals across different mTBI and treatment groups. Bars represent mean ± SEM. SHAM, control group; mTBI, mild traumatic brain injury group; TR, triamcinolone acetonide; NV, blank lipid nanocapsules; NT, TR-loaded lipid nanocapsules; d, day; h, hour; ±SEM, standard error of the mean. [file Image_1.tif]

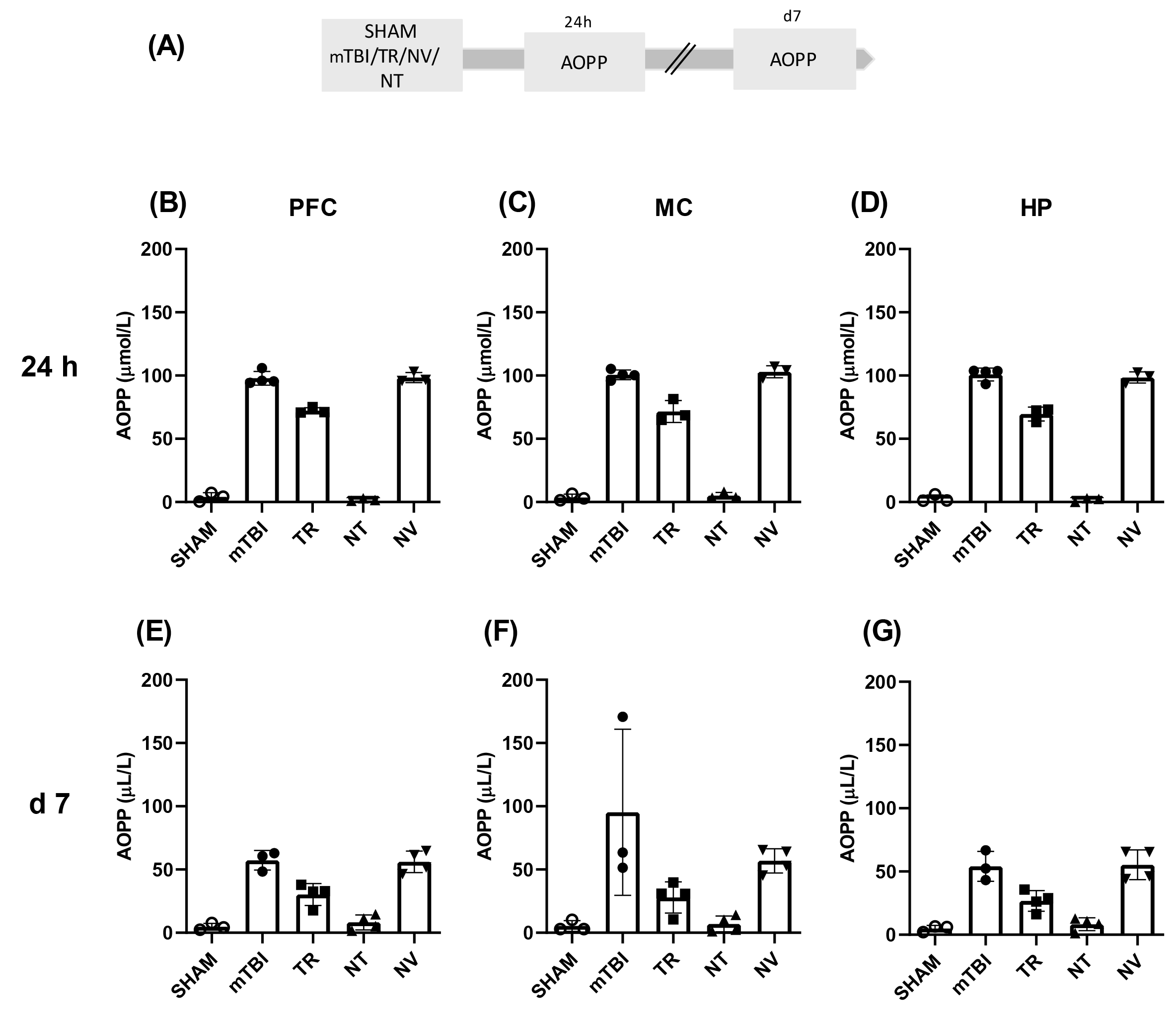

Supplement: Supplementary Figure 2 — Early treatment with NT reduced protein peroxidation induced by mTBI (A) Schematic representation of the experimental design for the quantification of AOPP, a marker of protein peroxidation. AOPP levels in distinct brain regions 24 h or 7 days after mTBI: (B,E) PFC, (C,F) MC, (D,G) HP. Bars represent the mean ± SEM. SHAM, control group; mTBI, mild traumatic brain injury group; TR, triamcinolone acetonide; NV, blank lipid nanocapsules; NT, TR-loaded lipid nanocapsules; d, day; h, hour; ±SEM, standard error of the mean. AOPP (advanced oxidation protein products), OS (oxidative stress), PFC (prefrontal cortex), MC (motor cortex), HP (hippocampus). [file Image_2.tif]

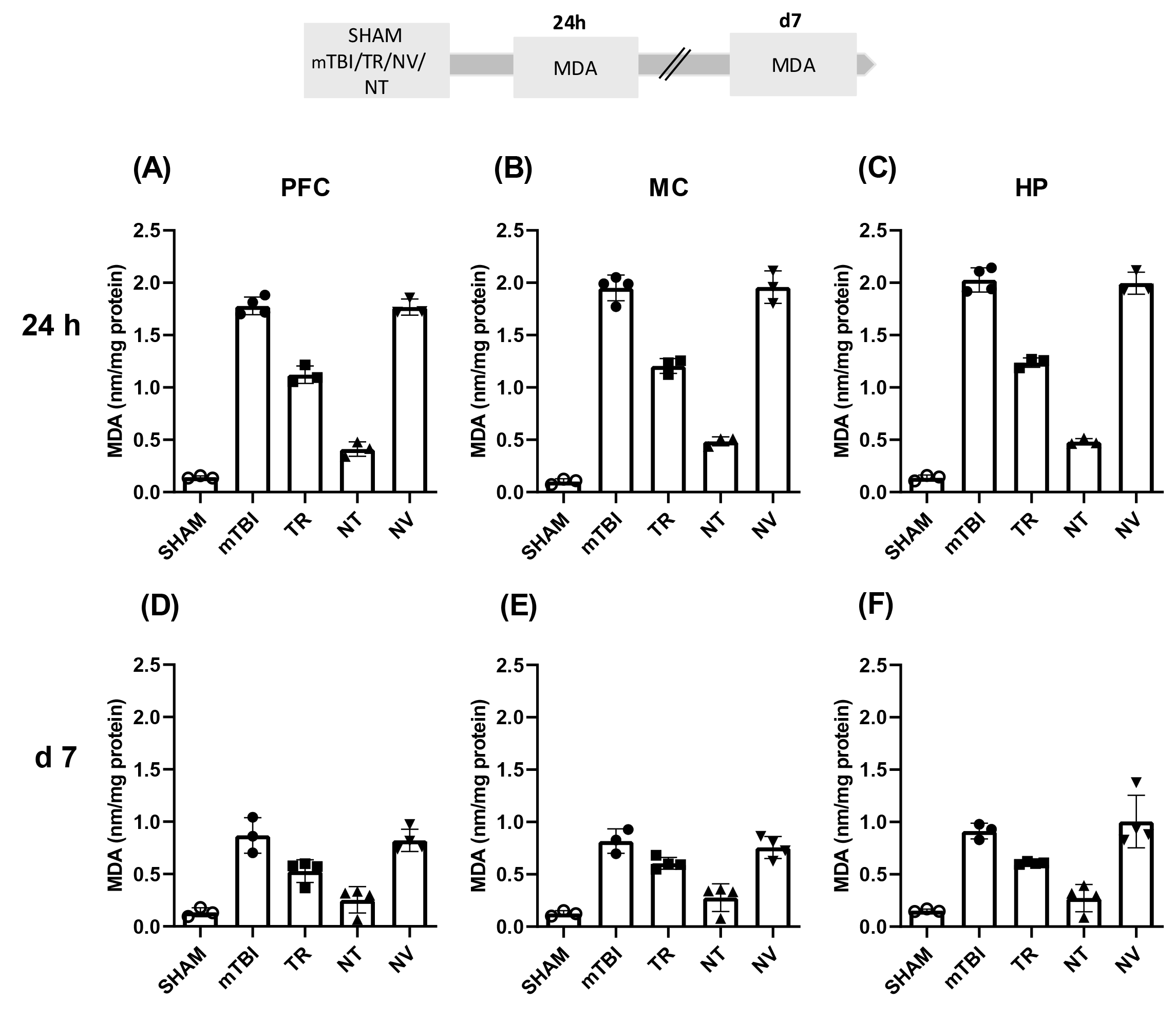

Supplement: Supplementary Figure 3 — Early treatment with NT reduced lipid peroxidation induced by mTBI (A) Schematic representation of the experimental design for the quantification of MDA, a biomarker of lipid peroxidation. MDA levels in different brain regions at 24 h or 7 days after mTBI: (B,E) PFC, (C,F) MC, (D,G) HP. Bars represent the mean ± SEM. SHAM, control group; mTBI, mild traumatic brain injury group; TR, triamcinolone acetonide; NV, blank lipid nanocapsules; NT, TR-loaded lipid nanocapsules; d, day; h, hour; ±SEM, standard error of the mean. AOPP (advanced oxidation protein products), OS (oxidative stress), PFC (prefrontal cortex), MC (motor cortex), HP (hippocampus). [file Image_3.tif]
